# The Cell Wall-Associated Mycolactone Polyketide Synthases Are Necessary but Not Sufficient for Mycolactone Biosynthesis

**DOI:** 10.1371/journal.pone.0070520

**Published:** 2013-07-23

**Authors:** Jessica L. Porter, Nicholas J. Tobias, Sacha J. Pidot, Steffen Falgner, Kellie L. Tuck, Andrea Vettiger, Hui Hong, Peter F. Leadlay, Timothy P. Stinear

**Affiliations:** 1 Department of Microbiology and Immunology, University of Melbourne, Victoria, Australia; 2 Department of Biochemistry, University of Cambridge, Cambridge, United Kingdom; 3 School of Chemistry, Monash University, Clayton, Victoria, Australia; 4 Department of Microbiology, Monash University, Clayton, Victoria, Australia; 5 Molecular Immunology Unit, Swiss Tropical and Public Health Institute, Basel, Switzerland; 6 University of Basel, Basel, Switzerland; French National Centre for Scientific Research - Université de Toulouse, France

## Abstract

Mycolactones are polyketide-derived lipid virulence factors made by the slow-growing human pathogen, *Mycobacterium ulcerans*. Three unusually large and homologous plasmid-borne genes (*mlsA1*: 51 kb, *mlsB*: 42 kb and *mlsA2*: 7 kb) encode the mycolactone type I polyketide synthases (PKS). The extreme size and low sequence diversity of these genes has posed significant barriers for exploration of the genetic and biochemical basis of mycolactone synthesis. Here, we have developed a truncated, more tractable 3-module version of the 18-module mycolactone PKS and we show that this engineered PKS functions as expected in the natural host *M. ulcerans* to produce an additional polyketide; a triketide lactone (TKL). Cell fractionation experiments indicated that this 3-module PKS and the putative accessory enzymes encoded by mup045 and mup038 associated with the mycobacterial cell wall, a finding supported by confocal microscopy. We then assessed the capacity of the faster growing, *Mycobacterium marinum* to harbor and express the 3-module Mls PKS and accessory enzymes encoded by mup045 and mup038. RT-PCR, immunoblotting, and cell fractionation experiments confirmed that the truncated Mls PKS multienzymes were expressed and also partitioned with the cell wall material in *M. marinum.* However, this heterologous host failed to produce TKL. The systematic deconstruction of the mycolactone PKS presented here suggests that the Mls multienzymes are necessary but not sufficient for mycolactone synthesis and that synthesis is likely to occur (at least in part) within the mycobacterial cell wall. This research is also the first proof-of-principle demonstration of the potential of this enzyme complex to produce tailored small molecules through genetically engineered rearrangements of the Mls modules.

## Introduction

**Figure 1:**
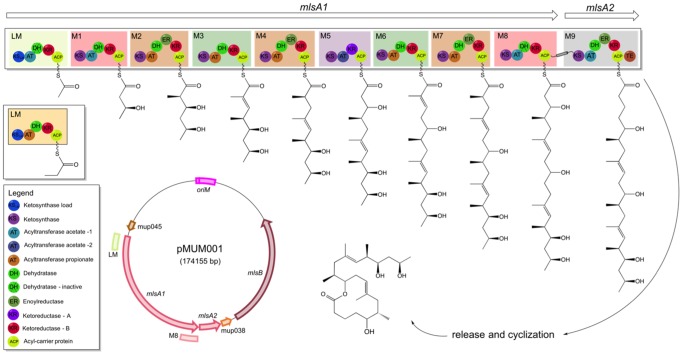
Overview of gene, module and domain organization of MlsA1 and MlsA2 from the mycolactone PKS, harboured by the plasmid pMUM001 from *M. ulcerans* Agy99 [10]. Depicted also is the proposed biosynthetic process for synthesis of the lactone core and upper side chain of mycolactone. Shown in inset (left) is the domain structure of the *mlsB* loading module from *M. ulcerans* Liflandii, showing the AT-propionate domain with propionate starter unit [13].

The bacterial pathogen *Mycobacterium ulcerans* causes disease through its ability to produce an immunomodulatory [1]–[8] macrocyclic polyketide called mycolactone [9]. Mycolactone synthesis depends on a highly unusual cluster of plasmid borne, type I modular polyketide synthases (PKS) (Figure 1) [10]. These large, multi-domain enzymatic complexes catalyze polyketide formation by the processive condensation of (usually) acetate and propionate subunits. Different combinations of reductive domains within the extension modules further modify the molecule at each extension step [11].

In the initial description of the mycolactone PKS (Mls) cluster, transposon mutagenesis showed that the *mls* genes were required for mycolactone synthesis [10]. However, it has never been shown that these genes are sufficient for toxin synthesis. There is only one published report of an experiment to test sufficiency, with the transfer (in two parts) of the 174 kb pMUM001 plasmid that harbours the *mls* genes to *Mycobacterium marinum*, a natural producer of many polyketide metabolites and a very close relative of *M. ulcerans* [12]. This experiment showed that the *mls* genes were expressed in *M. marinum* but mycolactones were not detected [12].

The mycolactone PKS modules and their constituent domains are encoded by three large genes (*mlsA1*: 51 kb, *mlsA2*: 7.2 kb, *mlsB*: 42 kb) that possess very high DNA sequence identity (Figure 1). For example, there is less than 3% nucleotide variation among all the ketosynthase (KS) domains of the 16 Mls extension modules [10], [13]. The high sequence repetition makes the *mls* locus prone to recombination-mediated deletion and it is common for laboratory passaged *M. ulcerans* to lose toxin production by this process [14]. *M. ulcerans* is a slow growing mycobacterium (>48 h doubling time) that is poorly transformable and for which there are few genetic tools. An additional complication is the host restriction of the pMUM plasmid. Studies of its *ori* showed plasmid replication within *M. marinum* but not within faster growing, but more distantly related, mycobacteria such as *M. smegmatis* and *M. fortuitum* [15]. These issues have made it difficult to explore the biosynthesis of mycolactones in detail.

Despite these barriers, some progress towards understanding mycolactone biosynthesis has been made by studying *M. ulcerans* strains that make different mycolactones. We originally speculated that the modules of the mycolactone PKS might be interchangeable; *i.e.* because domains are of near-identical sequence, they might be readily exchanged with each other to produce new module combinations and thus new polyketides, without the barriers that have evolved in other PKS where inter-domain identity is less than 80% and where tight specificity has evolved for native incoming precursor polyketides for a given module [10]. Support for this idea comes from the six naturally occurring mycolactone structural variants that have so far been described. These arise among different strains of *M. ulcerans* through in-frame recombination events within *mlsB* that swap, delete or duplicate MlsB modules and domains [16]–[24]. These genetic changes have resulted in a significant set of chemical modifications, including changes in the length, methylation state, hydroxylation pattern and stereochemistry of mycolactones. These changes also alter the biological activity of the molecule [4]. Such observations support our idea that the mycolactone PKS might be readily reprogrammed by genetically engineered module rearrangements to produce new, complex small molecules.

In the present study, we set out to develop a minimal trimodular version of the mycolactone PKS, reducing its size from 18 modules to only three, composed of a gene encoding the MlsA1 loading module, and module 8 and a second gene encoding module 9 from MlsA2. This trimodular arrangement was expected to produce a triketide lactone (TKL) when the genes were expressed in a compatible host bacterium. With this more tractable system we explored the potential of the Mls PKS for combinatorial polyketide biosynthesis as well as investigating Mls expression in different bacterial hosts and studying the cellular location of these enzymes within different host bacteria.

## Materials and Methods

### Bacterial Strains

*Escherichia coli* DH10B was cultured at 37°C in Luria-Bertani (LB) broth or LB agar. *M. marinum M* and *M. ulcerans* 06-3844 (the latter isolate also referred to as *M. marinum* 06-3844) [25] were cultured at 30°C in 7H9 Middlebrook broth or 7H10 Middlebrook agar as described [12]. Antibiotics were used at the following final concentrations in mycobacteria: apramycin 50 μg/mL; kanamycin 25 μg/mL; and hygromycin 50 μg/mL. The same concentrations of apramycin and kanamycin were used for *E. coli* and ampicillin was used at 100 μg/mL. All strains used are listed in Table 1.

**Table 1:** Bacterial strains used in the study.

Strain	Description	Reference
*E. coli*	DH10B	Invitrogen
**Mycobacteria**		
TPS8097	*M. ulcerans* 06-3844	[25]
TPS8164	*M. ulcerans* 06-3844+ pTPS331	This study
TPS8162	*M. ulcerans* 06-3844+ pTPS333	This study
TPS8307	*M. ulcerans* 06-3844+ pTPS438	This study
TPS8024	*M. marinum* ‘M’	[38]
TPS8256	*M. marinum* ‘M’+pTPS331	This study
TPS8254	*M. marinum* ‘M’+pTPS333	This study
TPS8313	*M. marinum* ‘M’+pTPS334+ pTPS629	This study
TPS8334	*M. marinum* M’+pTPS334+ pTPS629+ pTPS338	This study

### General DNA/RNA Methods

Methods for PCR, Sanger sequencing, ligation and cloning were as previously described [12]. *E. coli* and mycobacterial transformation methods were as previously described [12]. The primers used throughout are listed in Table S1. For RT-PCR, gene-specific primers were used to target mRNA within *mlsA1* LM-M8, *mlsA2*, mup038 and mup045. The *M. marinum crtI* gene was used as a positive control. Reverse transcription was performed by combining 1 μg of total RNA, 200 U of Superscript II reverse transcriptase (RT) enzyme (Invitrogen), 4 μl of 5x First Strand buffer (Invitrogen), 10 mM DTT (Invitrogen), 1 mM dNTPs (Promega), 2 pmol of gene-specific primer, made up to 20 μl with RNase-free-dH2O. After incubation at 42°C for 50 minutes, followed by a 15-minute heat inactivation at 70°C, the resulting cDNA was used as template in a standard PCR reaction. Reverse transcriptase reactions were also set up without the addition of RT enzyme to test for the presence genomic DNA.

### Plasmid Construction

Two mycobacterial expression plasmids were used. The first was pYUB412, modified to include the *mlsA1* promoter region [26] upstream of the unique PacI site in this vector, resulting in pTPS331. The second plasmid was based on replacing the hygromycin resistance gene, the L5 integrase gene, and attP site from pTPS331 with an apramycin resistance gene and pMUM001 *ori* as follows. The plasmid pTPS331 was digested with SacII to excise the hygromycin resistance gene and replace it with an apramycin resistance marker (pTPS404). A 6383 bp fragment spanning the pMUM001 *ori* and including *repA*, *parA*, and flanking NdeI (5′) and XbaI (3′) sites was PCR amplified from *M. ulcerans* Agy99 and cloned into AvrII/NdeI digested pTPS404, replacing the L5 integrase gene and attP site and resulting in the novel mycobacterial expression vector called pTPS628.

**Figure 2:**
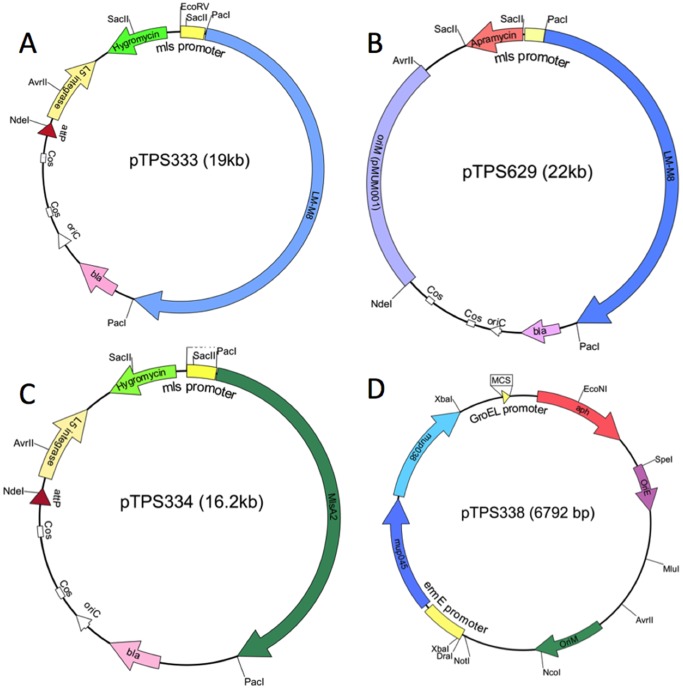
Schematic view of the mycobacterial expression vectors developed for this study. (A) pTPS333, pYUB412-based integrating vector with *mlsA1* LM-M8 under the control of the *mlsA1* promoter; (B) pTPS629, pMUM001-based low-copy number vector with *mlsA1* LM-M8 under the control of the *mlsA1* promoter; (C) pTPS334, pYUB412-based integrating vector with *mlsA2* under the control of the *mlsA1* promoter; (D) pTPS338 pMV261-based vector with mup038 and mup045 in an operon and under the control of the *ermE* promoter.

A truncated *mlsA1* gene comprising the loading module and module 8 (LM-M8) was constructed in *E. coli* DH10B by separate PCR amplification of each module and then cloning into pCDNA2.1, including the addition of 5′ and 3′ PacI sites respectively. The two modules were then translationally fused, using the BseRI site located between the ACP domain of the loading module and the KS domain of both module 1 and module 8 to create pTPS100. An 11 kb LM-M8 fragment was then excised from pTPS100 by PacI digestion and cloned into the unique PacI site downstream of the *mls* promoter in pTPS331 to create pTPS333 (Figure 2A). The same 11 kb fragment was also cloned from pTPS100 into the unique PacI site of the second mycobacterial expression vector pTPS628 to create pTPS629 (Figure 2B) (Table 2).

**Table 2:** Plasmids used in this study.

Name	Description	Source
pTPS207	pYUB412	[39]
pTPS331	pYUB412::P*_mls_*	This study
pTPS333	pYUB412::P*_mls_*:*mlsA1_*LM-M8*	This study
pTPS438	pYUB412::P*_mls_*:*mlsA1_*LM_P_-M8*	This study
pTPS334	pYUB412::P*_mls_*::*mlsA2*	This study
pTPS628	pYUB412::Apra::pMUM*ori*::P*_mls_*	This study
pTPS629	pYUB412::Apra::pMUM*ori*::P*_mls_*:: *mlsA1_*LM-M8	This study
pTPS338	pMV261::P*_ermE_*::mup045::mup038	This study

* LM-M8 refers to the loading module and module 8 regions encoded within *mlsA1*; LM_P_-M8 refers to the loading module from *M. ulcerans* Liflandii *mlsB* fused with *mlsA1* M8 from *M. ulcerans* Agy99.

A second version of the truncated *mlsA1* gene was also prepared, comprising the loading module from *mlsB* from *M. ulcerans* Liflandii with the *M. ulcerans* Agy99 *mlsA1* module 8 (LM_P_-M8). The *mlsB* loading module was PCR amplified from *M. ulcerans* Liflandii and modified to include flanking NotI and BseRI sites. This fragment was then ligated with *M. ulcerans* Agy99 MlsA1 M8 in pTPS099 using NotI/BseRI to create plasmid pTPS437, producing a translationally fused LM_P_-M8 in the same manner as above. The resulting 11 kb of DNA was excised from pTPS437 using PacI and cloned into the unique PacI site of pTPS331 to create pTPS438.

The full-length *mlsA2* gene was also cloned into pTPS331. The *mlsA2* gene was PCR amplified from *M. ulcerans* Agy99, modified to remove an internal NdeI site, and cloned into the NdeI/HindIII site of pET29, creating a C-terminal 5-histidine epitope tag for MlsA2 (MlsA2-His_5_). PacI sites were introduced to the 5′ and 3′ ends of *mlsA2-*His_5_ to permit excision and subcloning into the unique PacI site pTPS331 and resulting in the plasmid pTPS334 (Figure 2C) (Table 1).

A third expression vector harbouring the putative mycolactone accessory genes mup045 and mup038 under the P*_ermE_* promoter was also prepared by cloning a 2.3 kb fragment harbouring this promoter and these two genes as an operon into the unique XbaI site of pMV261 to create pTPS338 (Figure 2D).

### Stability Studies of pTPS629 in *M. marinum*

Late log-phase cultures of *M. marinum* M harbouring pTPS629 grown in the presence of apramycin, were diluted 1∶100 into three, 50 ml volumes of fresh 7H9 media without apramycin and incubation was continued at 30°C for 12 days. Aliquots of each culture were then removed at successive 3-day time points, appropriate dilutions were made and then plated on solid media with and without apramycin. Colonies were counted after ten days. The total cell number (expressed as colony forming units per ml) and the proportion of the total cell population that had maintained antibiotic resistance at each time point were calculated.

### Western Blotting

Mycobacterial whole cell lysates (WCLs) for Western immunoblotting were prepared as described [27]. WCLs were separated by SDS PAGE using NuPage Novex 3–8% Tris-Acetate polyacrylamide gels (Invitrogen). A primary polyclonal antibody was raised in rabbits against a recombinant form of the 34 kDa acyltransferase domain of the MlsA1 load module [28], then pre-adsorbed against WCL from *M. marinum* M and used at 1∶250 dilution. Primary polyclonal antibodies were raised in rabbits against the synthetic peptide (MRPINDIQVDGVPNC) derived from mup045. Mup045 antibodies were similarly pre-adsorbed against *M. marinum* WCL and used at a 1∶4000 dilution. As a secondary antibody, goat anti-Rabbit-IgG-HRP (Millipore) was used at a 1∶5000 dilution. Detection of MlsA2 was also achieved using a HRP conjugated anti-His antibody at a 1∶100 dilution (GenScript).

### Mass Spectrometry, Peptide Fingerprint Analysis

Bands from Coomassie-stained SDS-PAGE gels representing potential proteins of interest, were excised and treated with destaining solution (50% acetonitrile/50 mM tetra ethyl ammonium bicarbonate [TEAB]) at 37°C for 1 hour. Reduction of the sample was performed using 0.5 M Bondbreaker solution, diluted 1 in 10 in TEAB at 60°C for 1 hour, followed by alkylation using 100 mM iodoacetamide incubated at room temperature for 30 minutes. Iodacetamide was removed and the sample washed with 200 μl of destaining solution, followed by a 10-minute incubation in 50 ul of 100% acetonitrile. Once the gel slice became opaque and hard the acetonitrile was removed and the gel slice allowed to air dry at room temperature. In-gel trypsin digestion of the gel slice was performed overnight at 37°C (250 ng of trypsin made up in 50 μl of TEAB). Samples were analysed using an Agilent nanoCHIP 3D Ion Trap Mass Spectrometer and peptides identified using the MASCOT search engine (www.matrixscience.com).

### Bacterial Cell Fractionation

Cell fractionation was carried out as previously described [29]. Briefly, cells were harvested at 4,400×*g* and washed with 0.16 M NaCl. One gram of cells was resuspended in 1 ml of lysis buffer (0.05 M potassium phosphate buffer, 0.022% (v/v) β-mercaptoethanol, pH 6.5) containing 2.4 mg/ml lysozyme and incubated at 37°C for at least two hours. Cells were disrupted using a Precellys24 tissue homogenizer (Bertin Technologies) at speed 6500, twice for 45 sec. Unbroken cells were removed by centrifugation at 1000×*g* for 5 mins. Lysates were subjected to ultracentrifugation at 27,000×*g* twice for 40 mins at 4°C to isolate the cell wall (P27) fraction. The supernatant was then centrifuged at 100,000×*g* for 1 hr at 4°C to separate into the membrane (P100) and cytosolic (SN100) fractions.

### Immunofluorescence Assay

Bacteria were fixed on glass slides and stained using the anti-mup045 and anti-AT domain polyclonal sera described above as primary antibodies These antibodies were applied at 1∶1000 dilution in PBS with 0.1% (v/v) Tween 20 for 1 h at 20°C. Alexa Fluor 568 (Invitrogen) conjugated goat anti-rabbit immunoglobulin G was used as secondary antibody at 1∶200 dilution for 1 h at 20°C. Cells were mounted in ProLong Gold anti-fade reagent containing 4′,6-Diamidino-2-phenylindole (DAPI; Invitrogen). Images were acquired using a Zeiss LSM700 confocal laser-scanning microscope (x100 oil immersion objective) and images were processed using Zen software (Zeiss, 2009).

### TKL Isolation and LC-MS Analysis

Mycobacteria were grown for 4 weeks on solid media and 2 cm^2^ of colony material and surrounding agar were excised and potential triketide lactones (TKLs) were extracted with ethylacetate. Briefly, colony material and surrounding agar was placed into a screw-cap tube containing 500 μl of 100 μm glass beads and 1 mL of a ethylacetate/formic acid mixture (1.2 ml ethylacetate +20 μl of formic acid), samples were placed at 50°C for 15 minutes before bead-beating, 3×45 second pulses at speed 5 in a Precellys24 tissue homogenizer, the samples were then briefly centrifuged to collect the liquid phase. The resulting extracts were dried down with N_2_, the residue was dissolved in 200 μl HPLC grade methanol and 30 μl of the sample was analysed by liquid chromatography-mass spectrometry (LC-MS) using a LTQ mass spectrometer (Thermo Finnigan), with positive mode electrospray ionisation. The mass spectrometer was coupled with a HP1200 HPLC system (Agilent) fitted with a Phenomenex Prodigy C18 column (5 μm, 2.0×250 mm). Samples were eluted with acetonitrile and MilliQ water with 0.1% formic acid using a gradient of 5% to 50% acetonitrile over 25 min at a flow rate of 300 μl/min. Production of triketide lactone was judged by comparison with the standard (5-hydroxyhexanoic acid lactone, Alfa Aesar, UK) using on-line LC-MS/MS analysis on [M+H]+ ion at m/z 115.2 with normalized collision energy of 20%.

## Results

### Expression of Truncated MlsA1 PKS in *M. ulcerans*

**Figure 3:**
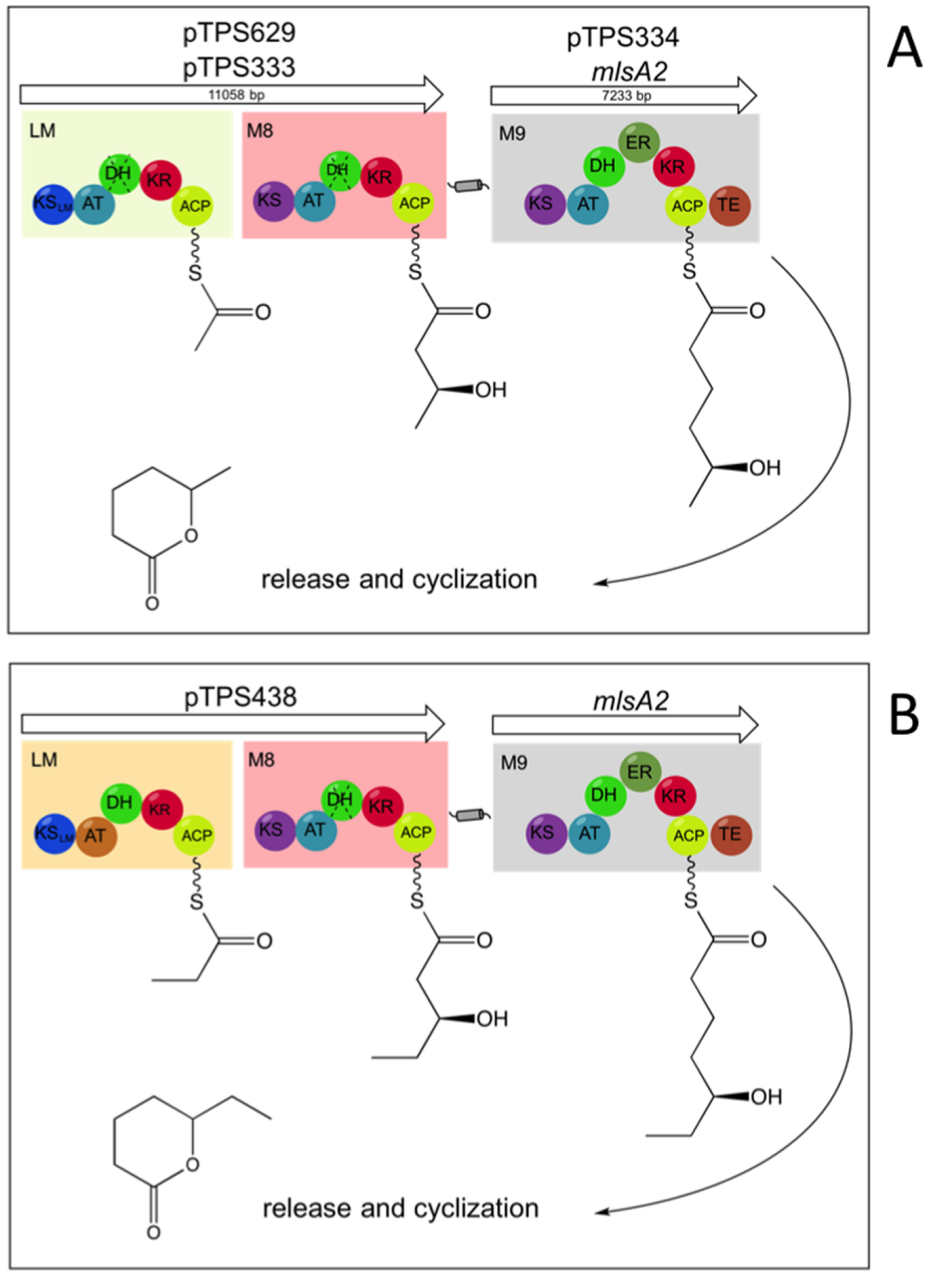
Gene, module and domain organisation in the bimodular MlsA-derived PKS. (A) Arrangement using the MlsA1 load module from *M. ulcerans* Agy99 for the biosynthesis of methyl-triketide lactone; (B) Arrangement using the MlsA1 load module from *M. ulcerans* Liflandii for the biosynthesis of ethyl-triketide lactone.

**Figure 4:**
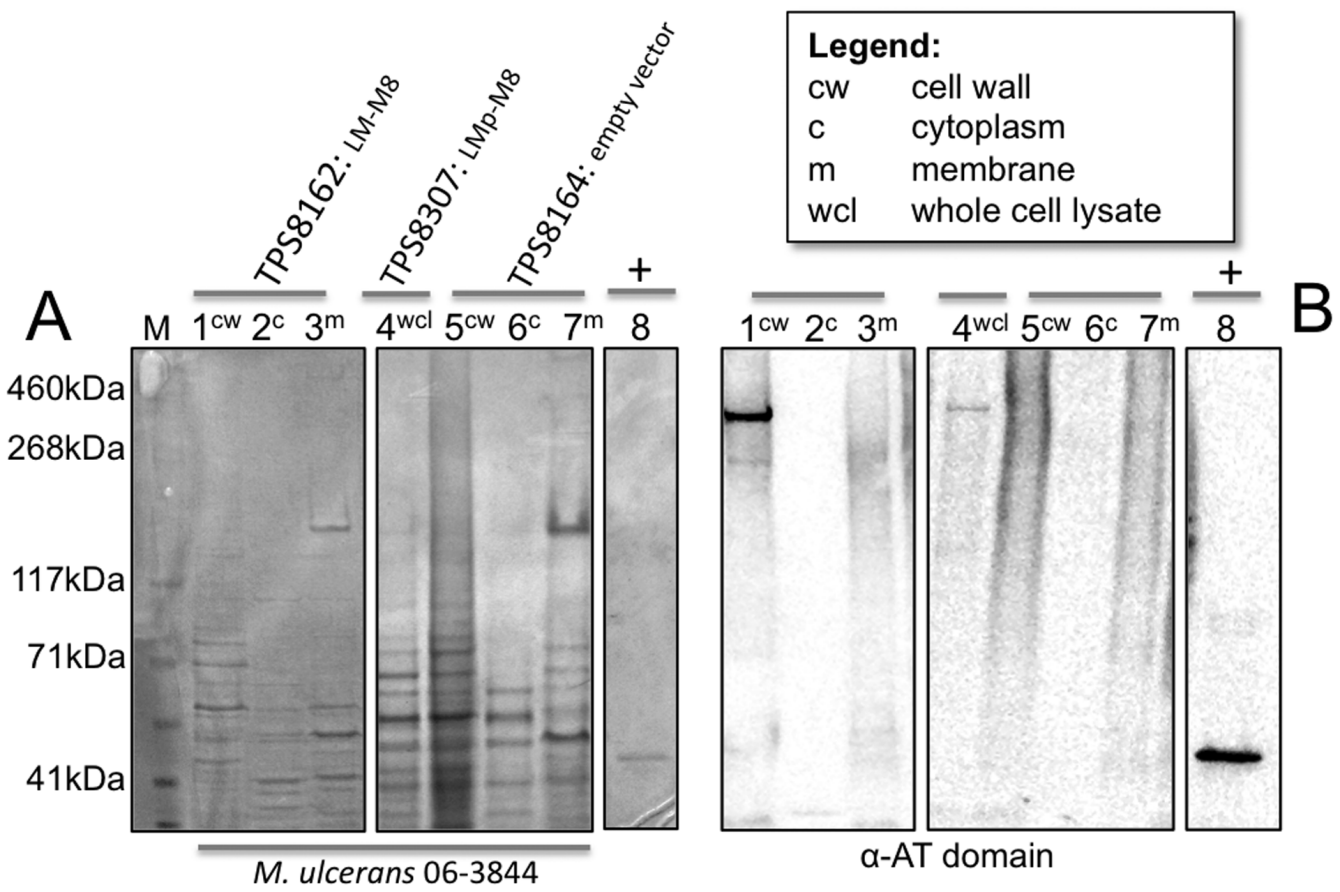
SDS-PAGE and western immunoblot analysis of *M. ulcerans* 06-3844 expressing TKL constructs. (A) SDS-PAGE separation and Coomassie-stained protein of 10 μg of *M. ulcerans* 06-3844 containing *mlsA1* LM-M8 (TPS8162), LM_P_-M8 (TPS8307) or empty vector (TPS8164) cell fractions (B) Western immunoblot analysis of (A) with an anti-AT domain antibody showing the presence of a ∼400 kDa protein produced by *M. ulcerans* harbouring either LM-M8 or LM_P_-M8 (lane 1 and 4). Positive control is purified, recombinant acyltransferase derived from MlsA2.

MlsA1 is encoded by a single 51 kb gene and is composed of a loading module and eight extension modules (Figure 1). The large size of *mlsA1* and its significant internal sequence repetition - essentially composed of eight, 6 kb direct repeats - make it difficult to modify and mobilize. We therefore constructed a small, more manageable *mlsA1* gene, comprising only the loading module and module 8 (LM-M8) and we cloned it into the *Mycobacterium/E. coli* shuttle vector pYUB412 that was also modified to include the native *mlsA1* promoter upstream of LM-M8 (pTPS333). A second LM-M8 construct called LM_P_-M8 (pTPS438) was also prepared, taking the loading module from *M. ulcerans* Liflandii, where the acyltransferase domain of the loading module confers specificity for methylmalonyl-CoA (propionate instead of acetate starter unit) [13], [20]. For each construct, the resulting 11 kb CDS was predicted to encode a 390 kDa protein (Figure 3). When plasmid pTPS333 and pTPS438 were separately transferred to *M. ulcerans* strain 06-3844, a strain that is more amenable to genetic manipulation than *M. ulcerans* Agy99 and a natural producer of mycolactone F, we observed by Western immunoblot (using an antibody raised against the acyltransferase domain of the MlsA1 loading module) the presence of a protein with a predicted mass around 400 kDa in each strain that was absent from the same strain harbouring the empty vector (Figure 4). Cell fractionation was also performed for *M. ulcerans* TPS8162. Interestingly, the LM-M8 Mls protein and a faint band at ∼260 kDa, likely representing the native MlsA2, were detected exclusively in the cell wall fraction, suggesting the mycolactone PKS might be cell wall associated. Although the same amounts of protein were loaded (Figure 4A), the ∼260 kDa band representing MlsA2 was not detected in TPS8307 (LM_P_-M8) or TPS8164 (empty vector) (Figure 4B).

### Production of the Predicted TKLs in *M. ulcerans*

**Figure 5:**
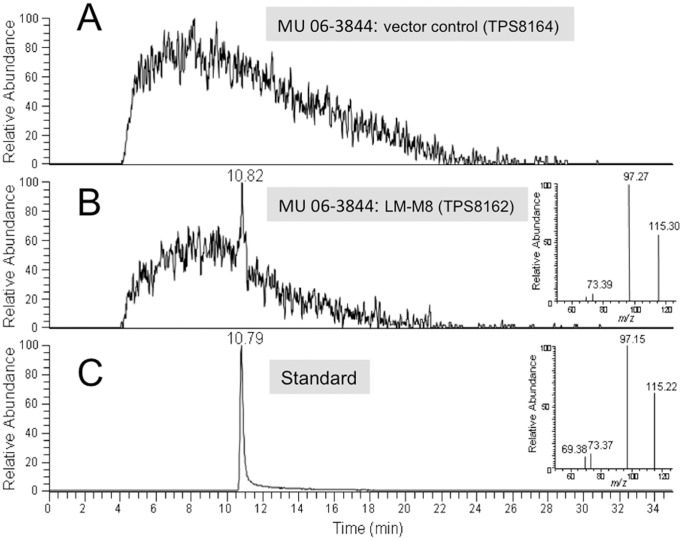
Chromatogram of fragment ions at m/z 73.3 and 97.3 from MS/MS analysis of [M+H]+ ion at m/z 115.1. (A) control; (B) sample and (C) standard compound, 5-Hydroxyhexanoic acid lactone. Insets are the MS/MS spectrum for [M+H]+ ion at m/z 115.

We expected that the C-terminal docking domain present in Module 8, expressed from pTPS333, would link with its cognate N-terminal docking domain of the endogenous MlsA2 (Module 9) enzyme to produce a functional PKS, capable of synthesizing the two predicted TKLs (Figure 3). *M. ulcerans* strain 06-3844 harbouring pTPS333 (TPS8162) or pTPS438 (TPS8307) was cultured for 4 weeks, after which bacterial cells were harvested and subjected to ethylacetate extraction. Analysis of the extracts by HPLC-MS (Figure 5) revealed that TPS8162 produced, in addition to mycolactone F, the expected methyl triketide lactone (5-hydroxyhexanoic acid lactone), albeit in low yield. The yield of mycolactone F was not significantly diminished compared to controls (data not shown). In contrast, the predicted ethyl TKL (5-hydroxyheptanoic acid lactone) was not detected in the second recombinant *M. ulcerans* strain TPS8307 (data not shown).

### The Truncated MlsA1 and Full-length MlsA2 PKS are Expressed in *M. marinum*

We next tested the capacity of *M. marinum*, a faster growing and genetically more tractable close relative of *M. ulcerans*, to express LM-M8 and *mlsA2* to reconstitute a 3-module PKS, capable of synthesizing TKL as above (Figures 3–5). To attempt this experiment we first cloned the full-length 7.2 kb *mlsA2* gene, under the control of the *mlsA1* promoter into our pYUB412-based expression vector, pTPS331, to create pTPS334 (Figure 2C).

**Figure 6:**
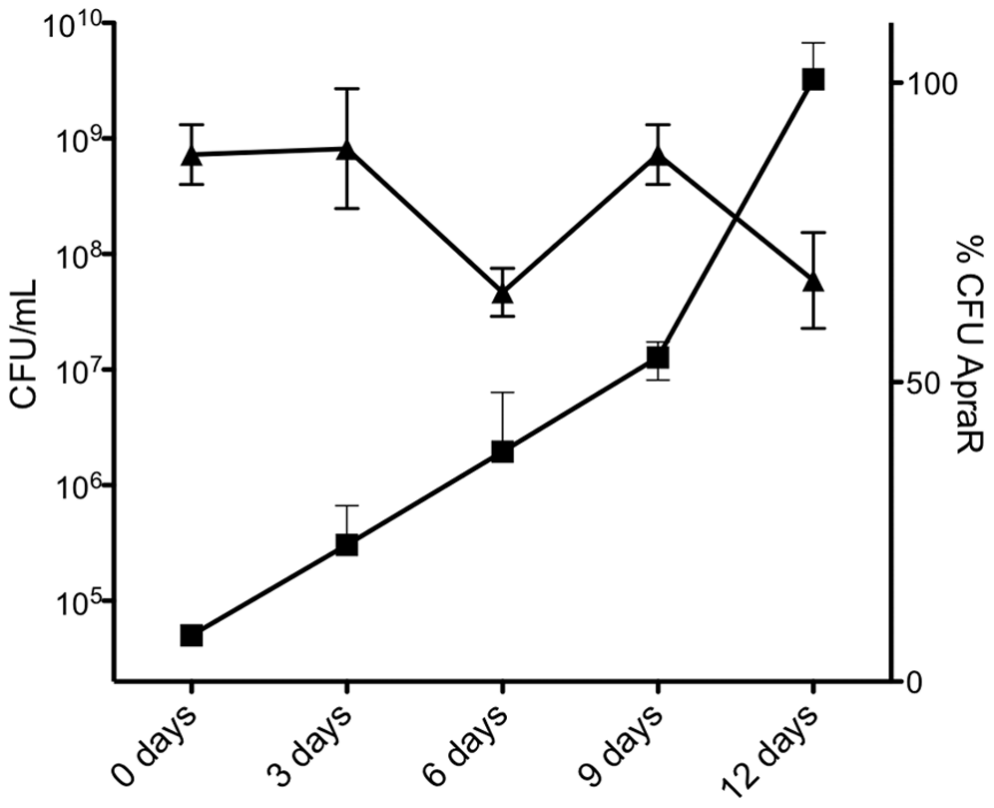
Stability of pTPS629 in *M. marinum* M cultured in the absence of apramycin antibiotic selection. A late log-phase culture of *M. marinum* harbouring pTPS629 (and *mlsA2* on pTPS334) grown in the presence of apramycin and hygromycin, was shifted to media without apramycin and then monitored at successive time points by determining the cfu/ml on media with the antibiotic (squares) and calculating the percentage of *M. marinum* cells retaining apramycin resistance (right hand Y-axis, triangles). These results depict the mean and standard deviation of at least biological triplicates.

To also introduce the LM-M8 construct into the same host strain, we developed a pTPS331-compatible mycobacterial expression vector called pTPS628 (refer methods, Figure 2B) based on the *M. ulcerans* pMUM001 mycolactone plasmid (Figure 1). This plasmid is maintained at 1–2 copies per cell and has a restricted host range, replicating in *M. marinum* but not in fast-growing mycobacteria such as *M. smegmatis* and *M. fortuitum* [10], [15]. We cloned LM-M8 into this vector (pTPS629) and tested the stability of the plasmid in *M. marinum* in the absence of antibiotic selection. Approximately 70% of the population retained pTPS629 in the absence of apramycin selection over the course of the 12-day growth curve experiments (Figure 6).

**Figure 7:**
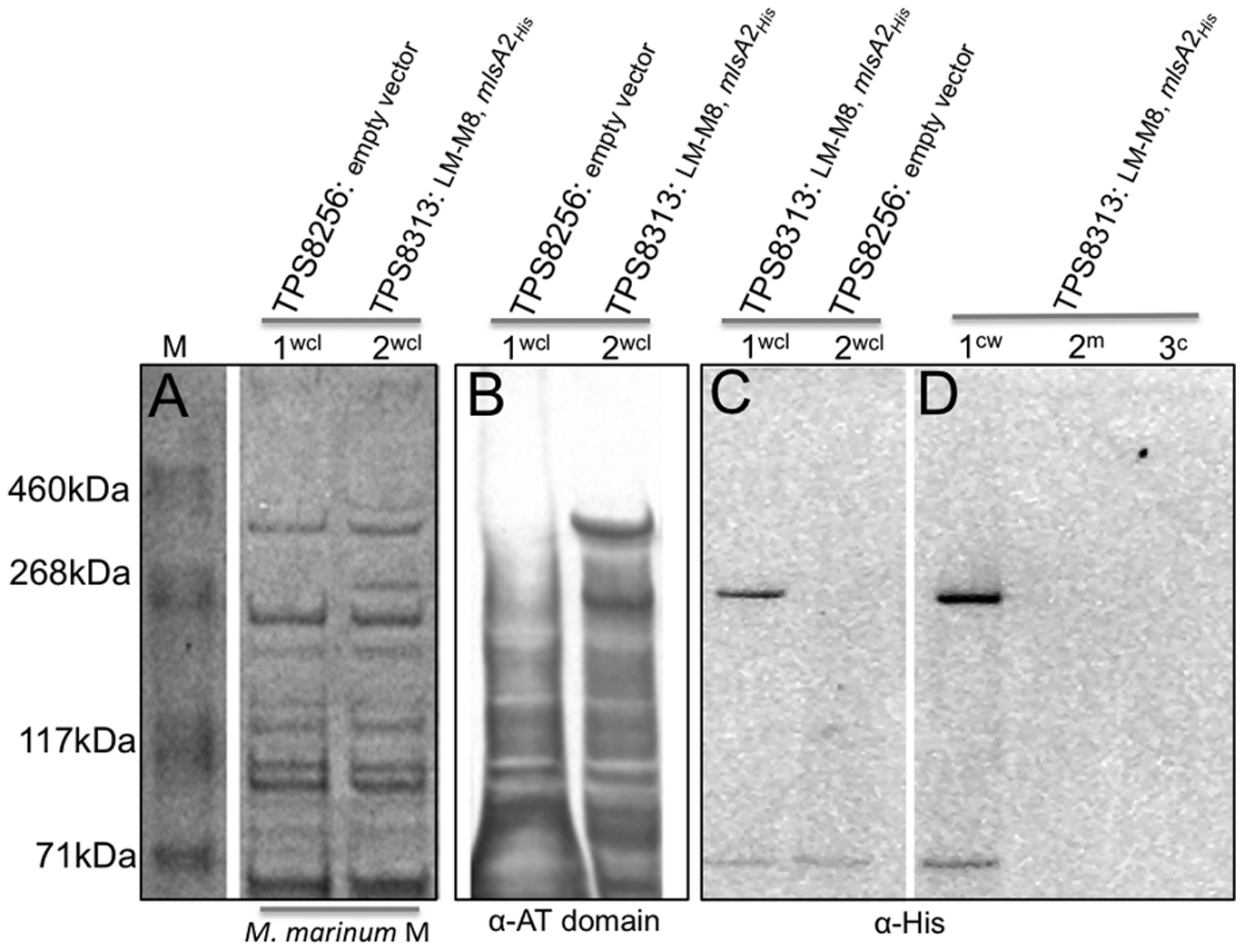
SDS-PAGE and western immunoblot analysis of whole cell lysates and cell fractions of *M. marinum* M harbouring plasmids expressing LM-M8 and MlsA2_His_. (A) SDS-PAGE separation and Coomassie-stained protein gel of 10 μg of whole cell lysate of *M. marinum* M with LM-M8 and *mlsA2* (TPS8313) and empty vector control (TPS8256); (B) Western immunoblot of (A) using an anti-AT domain antibody; (C) Western immunoblot of (A) using an anti-His antibody; (D) Western immunoblot of cell fractions from *M. marinum* M harbouring plasmids expressing LM-M8 and MlsA2_His_ using an anti-His antibody, showing MlsA2 is present only in the cell wall (P27) fraction. The reactivity of the anti-His antibody to a protein with a mass ∼65 kDa in panels (C) and (D) is the known cross-reactivity with the polyhistidines of mycobacterial GroEL (Hsp65) [37].

*M. marinum* was transformed with pTPS334 (*mlsA2*) and pTPS629 (*mlsA1* LM-M8) and whole cell lysates were screened by Western immunoblot, with the acyltransferase domain antibody. Two proteins with masses around 400 kDa and 260 kDa were observed, corresponding to MlsA2 and LM-M8 respectively (Figure 7A,B). We took advantage of the C-terminal His_5_ tag introduced into MlsA2 and also screened the whole cell lysates with an anti-His_5_ antibody. As expected, a protein with a mass of 260 kDa was detected (Figure 7C), that was confirmed by peptide mass fingerprinting, with five high scoring peptides (*p*<0.05) spanning the 2416 aa of the MlsA2 polypeptide (positions 75–91, 1286–91, 1631–64, 1864–94, 1948–63, 2362–79). Cell fractionation was also performed and the Western blotting repeated, suggesting again that MlsA2 associates with the mycobacterial cell wall (Figure 7D).

### The Predicted TKL is not Produced in Recombinant *M. marinum*

**Figure 8:**
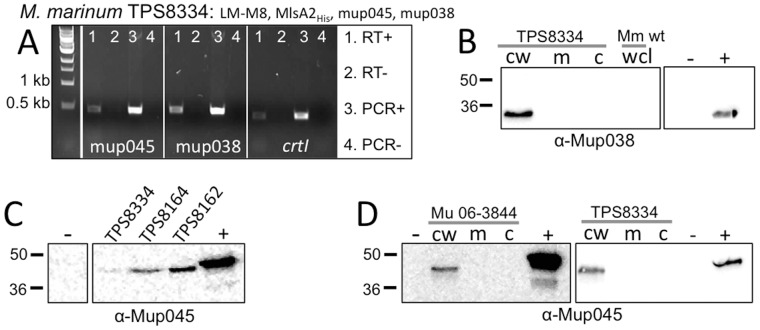
Expression of mycolactone accessory enzymes encoded by mup045 and mup038. (A) RT-PCR of mup045, mup038 and *crtI* on RNA extracted from *M. marinum* M containing plasmids expressing LM-M8 and mup045-mup038 under control of the P*_ermE_* promoter. Western immunoblots showing: (B) presence of Mup038 in the cell wall fraction of *M. marinum* TPS8334; (C) presence of Mup045 in whole cell lysates from *M. marinum* M expressing LM-M8 and mup045-mup038 (TPS8334) as well as *M. ulcerans* 06-3844 harbouring pTPS331 (TPS8164) or pTPS333 (TPS8162); (D) Localization of Mup045 to the cell wall in *M. ulcerans* 06-3844 wild type and *M. marinum* TPS8334 cell fractions, showing reactivity against Mup045 in all strains, and localized to the cell wall fraction in both strains. Positive controls are purified, recombinant Mup038 and Mup045. (D).

*M. marinum* with pTPS334 (MlsA2) and pTPS629 (LM-M8) was expected to produce the same TKL previously detected in *M. ulcerans* 06-3844. However, LC-MS analysis of ethylacetate extracts from this recombinant *M. marinum* failed to detect the expected metabolite (data not shown). The genes mup045 and mup038 cluster with the *mls* genes on pMUM001 and are predicted to encode accessory enzymes, important for mycolactone synthesis (Figure 1) [10]. A third expression vector was prepared containing mup045 and mup038 as an operon under the control of the constitutive Streptomyces *ermE* promoter (pTPS338) and used to transform *M. marinum* already harbouring LM-M8 and *mlsA2*. RT-PCR analysis of the triple plasmid recombinant strain (TPS8334) showed that these two additional genes were expressed (Figure 8A). Western blots using a peptide-derived polyclonal antibody against mup038 in *M. marinum* TPS8334 fractions suggests Mup038 also localizes to the cell wall (Figure 8B). Mup038 immunoblots with *M. ulcerans* 06-3844 wild type showed very faint reactivity to the antibody, suggesting low expression of Mup038 in the natural host (data not shown). Immunoblotting with polyclonal antibodies raised against a Mup045-specific peptide confirmed Mup045 protein expression in wild type *M. ulcerans* 06-3844 (Figure 8C). Fractionation experiments with wild type and recombinant *M. ulcerans* 06-3844 showed endogenous Mup045 partitioning with the cell wall fraction (Figure 8D).

**Figure 9:**
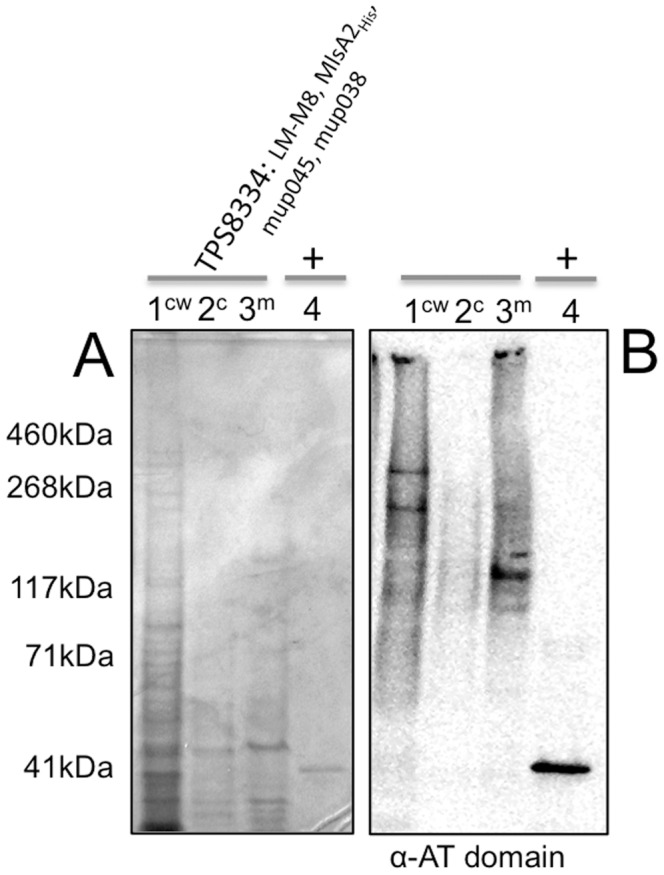
Expression analysis of recombinant *M. marinum* TPS8334. (A) Coomassie stained SDS-PAGE and (B) Western immunoblot of cell wall fractions of *M. marinum* expressing LM-M8, MlsA2_His_, Mup038 and Mup045 (TPS8334) using an anti-AT domain antibody demonstrating the presence of the heterologously expressed PKSs in the cell wall fraction of this strain. The positive control is purified, recombinant acyltransferase from MlsA2.

The same fractions from *M. marinum* TPS8334 were also screened to confirm the presence and location of the MlsA1 LM-M8 PKS and MlsA2. Immunoblotting with the α-AT domain antibody showed again that LM-M8 and MlsA2 associate with the mycobacterial cell wall (Figure 9). Together with the other fractionation experiments for recombinant *M. marinum* and *M. ulcerans* 06-3844 (Figures 4 & 7), these data suggest that mycolactone biosynthesis occurs in association with the mycobacterial cell wall. Ethylacetate extracts were then prepared from the recombinant *M. marinum* and screened for TKL by LC-MS, but disappointingly the expected metabolite was not detected in the heterologous host.

#### Fluorescence microscopy confirms a cell wall location for Mup045

**Figure 10:**
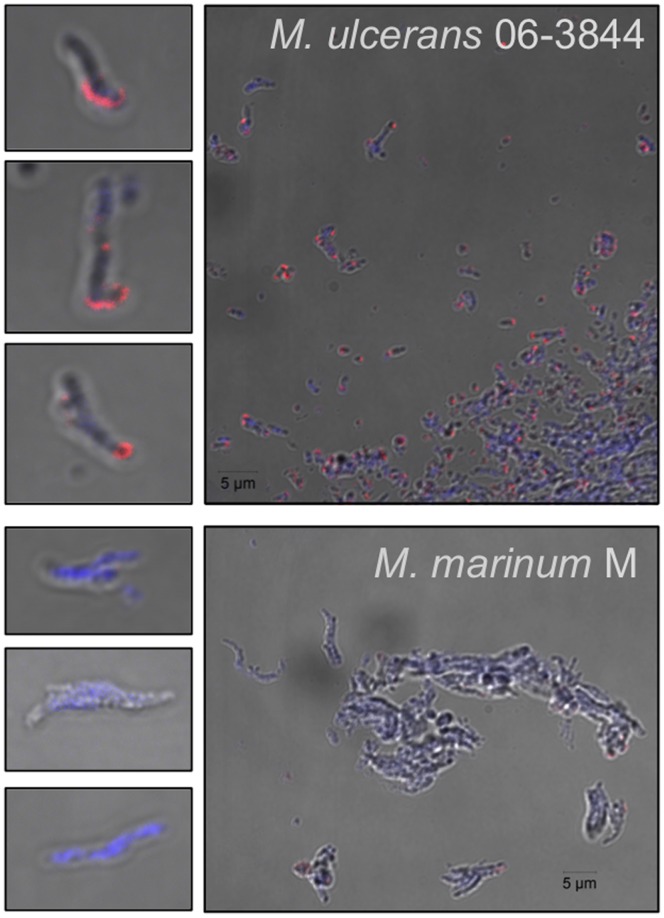
Imaging of Mup045 in association with the mycobacterial cell wall in *M. ulcerans* 06-3844 wild type compared with *M. marinum* M wild type, as revealed by DIC fluorescence microscopy. Cells were stained with DAPI and incubated with a primary anti-Mup045 antibody with visualization by a secondary antibody conjugated to Alexa fluor-568.

Taking advantage of the specificity of the Mup045 antibody, we performed fluorescence microscopy to test our proposition that the mycolactone machinery is cell wall associated (Figure 10). A distinctive, focal, pericellular pattern of fluorescence was observed in *M. ulcerans* that was absent from *M. marinum* (Figure 10). These observations provide further support for a cell wall location of Mup045. We also attempted microscopy with the α-AT domain antibody, however, as seen with the Western immunoblots (Figure 7), this antibody was somewhat cross-reactive and stained *M. ulcerans* and *M. marinum* equally well (data not shown).

## Discussion

In this study we have developed two modified mycolactone PKS genes and used them to gain a better understanding of the multienzymes they encode, as well as explore their potential for combinatorial polyketide biosynthesis. In the first instance we constructed a minimum version of the 51 kb *mlsA1* gene (LM-M8). When introduced into *M. ulcerans* via a pYUB412-based integrating mycobacterial expression vector, the *mlsA1* LM-M8 produced the expected triketide lactone by comparison with the authentic compound and high-resolution mass spectrometry analysis (observed 115.0755, calculated for C_6_H_11_O_2_^+^[M+H]^+^115.0754) (Figures 4 & 5). This experiment showed *mlsA1* LM-M8 formed a functional PKS and demonstrated in principle that the *mls* genes can indeed be manipulated to produce novel small molecules, in this case a methyl triketide lactone (TKL). However, the amount of the methyl TKL obtained was low and when a second construct was tested using the same vector in the same *M. ulcerans* strain, but where the AT-acetate domain of the loading module was replaced with an AT-propionate version, the expected protein subunit was present (Figure 4), but the predicted ethyl TKL was not.

There are several possible reasons for the low yield or absence of detectable TKL. It may be that the chain-terminating thioesterase (TE) of MlsA2 discriminates against the shorter chains or that transfer of the normal mycolactone F octaketide intermediate onto the endogenous MlsA2 outcompetes transfer of the diketide acyl chain from the truncated hybrid PKS (Figures 1 & 2).

To try and test the latter hypothesis we transformed *M. marinum* M with *mlsA1* LM-M8 and an epitope tagged version of *mlsA2*. *M. marinum* is potentially an ideal host to test expression of the *mls* PKS genes. It is very closely related to *M. ulcerans*, but does not contain the pMUM plasmid and does not make mycolactones. The *M. marinum* genome encodes a substantial secondary metabolome that includes many type 1 PKS loci and the necessary accessory enzymes required by these mega-enzymes such as 4′-phosphopantetheinyl transferase to transfer a 4′-phosphopantetheinyl (4′-PP) moiety from coenzyme A (CoA) to the ACP [30]. To facilitate independent expression of two, relatively large genes in *M. marinum* (*mlsA1* LM-M8: 11 kb and *mlsA2*: 7.2 kb) we also developed a novel expression plasmid based on the pMUM001 *ori*. This plasmid has an active *par* locus and is stably maintained at low copy number even in the absence of antibiotic selection in *M. marinum* (Figure 6) [10], [15]. The *mlsA1* LM-M8 gene was cloned into this pMUM-based plasmid and this construct, together with *mlsA2* cloned into pYUB412 was used to transform *M. marinum* M. Each plasmid expressed their respective *mls* PKS genes from the *mlsA1/mlsB* promoter; a regulatory sequence we have previously shown to function in *M. marinum* M [26]. Immunoblotting confirmed the presence of both MlsA1 LM-M8 and MlsA2 in *M. marinum* (Figure 7B). The identity of MlsA2 was additionally confirmed by immunodetection of its C-terminal 5x His epitope (Figure 7C). However, while the Mls PKS appeared to be expressed at reasonable levels (proteins visible by Coomassie staining), this recombinant *M. marinum* did not produce detectable methyl TKL, even in the absence of competing mycolactone intermediates. Supplying *M. marinum* with the mycolactone accessory enzymes encoded by mup045 and mup038 on pMUM001 did not activate TKL synthesis. Mup045 resembles an atypical acyltransferase and Mup038, a type II thioesterase with a proposed role in ensuring the processivity of the Mls system [10].

The absence of the expected TKL prompted us to examine the cellular distribution of the Mls proteins. Immunoblotting showed co-partitioning of the Mls PKS and mup045 with the mycobacterial cell wall components in both *M. ulcerans* 06-3844 and the recombinant *M. marinum* TPS8334 (Figures 4, 8 &9). Very low levels of Mup038 were detected in wild type *M. ulcerans* strains (data not shown), consistent with our previous study that showed low mup038 promoter activity compared to *mlsA1, mlsB* and mup045 [26]. Fluorescence microscopy also supported a cell wall location for the mycolactone synthesis machinery, with Mup045 present in distinct foci around the *M. ulcerans* cell wall (Figure 9). Attempts to similarly visualize the Mls PKS were not successful due to a lack of specificity with our anti-AT serum. This serum preparation was produced using the entire AT domain, a domain widely present in mycobacterial PKS and fatty-acid synthases (e.g. in *M. marinum* there are 16 proteins with this domain, sharing >50% aa sequence similarity). Future research to explore our initial observations will require Mls PKS antibodies with improved specificity. Nevertheless, the data we present here suggest that mycolactone synthesis is occurring at or within the cell wall, consistent with previous reports describing mycolactone blebbing from the bacterial cell in lipid and protein-rich vesicles [31] and an *M. ulcerans* proteomic investigation that also detected the Mls PKS in the cell wall [32]. It is interesting to speculate how these megadalton-sized molecular machines with their predicted homodimeric and interconnected structure are arranged within the mycobacterial cell wall. Only a handful of reports have uncovered PKS linked to the cell wall [33], [34]. In mycobacteria there is a proposed pathway linking an RND superfamily transporter protein (MmpL7) with the type I PKS, PpsE [34]. It is possible that the Mls system is similarly dependent on MmpL-like proteins for mycolactone export.

A recent comparative genomic study of the evolution of *M. ulcerans* has revealed that cell wall-associated genes are undergoing significant diversifying selection compared to *M. marinum* [35]. Maybe this evolutionary signature is a response to selective pressures that are shaping *M. ulcerans* to accommodate the mycolactone synthesis machinery. Furthermore, it is possible that some of these changes are essential for the correct positioning, location, and thus functioning of the Mls PKS within the mycobacterial cell wall. *M. marinum*, despite its high shared genetic identity with *M. ulcerans*, may therefore not have the requisite genetics and cell wall composition for the Mls PKS to function correctly.

Our findings illustrate the promise of the Mls PKS for combinatorial polyketide biochemistry but also underline the experimental challenge. While a minimal Mls PKS produced the expected TKL, yields of metabolite were low or undetectable. To better understand the limits of modularity in this remarkable system, the next step should be direct assessment of the activity and specificity of individual *mls* domain and module activity, using recombinant proteins and diverse synthetic substrates, as recently described for a fully reducing module of the nanchangmycin type I PKS [36].

Meanwhile, the Mls and accessory enzyme expression constructs we have developed here have provided intriguing additional evidence that, in mycolactone formation at least, polyketide assembly on the multienzyme modular PKS and subsequent tailoring and export are carried out in intimate association with the cell wall.

## Supporting Information

Table S1 Oligonucleotides used in this study.
(DOCX)
