# Supplementary material for: The Cell Wall-Associated Mycolactone Polyketide Synthases Are Necessary but Not Sufficient for Mycolactone Biosynthesis
Source: PLoS One. 2013 Jul 23;8(7):e70520. doi: 10.1371/journal.pone.0070520 (PMC3720922; doi:10.1371/journal.pone.0070520)
Supplement: Table S1 — Oligonucleotides used in this study. (DOCX) [file pone.0070520.s001.docx]

**Table S1:** Oligonucleotides used in this study

| **Primer #** | **Oligonucleotide (5’-3’)^*^** | **Function** |
| --- | --- | --- |
| 612 | CGC**GATATC**CAAGCGCTGCGACAGT | EcoRV-703-F (to PCR up Pmls from Agy99 incorporating EcoRV site at 5’ end) |
| 556 | GTCTAAATTCCAACGTGCAG | LM sequence out (used with EcoRV-703-F to PCR up Pmls from Agy99, has native PacI site within this region to use for cloning) |
| 1854 | GGAATTC**CATATG**cgaaatgccaagacctcag | pMUM-F-NdeI (for pYUB412:Apra:pMUMori:Pmls:C1) |
| 1855 | GC**TCTAGA**cgcggagcctaacatctct | pMUM-R-XbaI (for pYUB412:Apra:pMUMori:Pmls:C1) |
| 706 | TCC**CCGCGG**TTCATGTGCAGCTCCATCAGC | Apra_SacII_F (for pYUB412:Apra:pMUMori:Pmls:C1) |
| 707 | TCC**CCGCGG**CATTGAGCGTCAGCATATCAT | Apra_SacII_R (for pYUB412:Apra:pMUMori:Pmls:C1) |
| 221 | ccactgagatccggctg**ttaattaa**gcccgaaaggaagc | Quikchange oligos for altering the 3’ end of the M9 cassette in Pet29 to incorporate a PacI site (Mod9-PacIend-F) |
| 222 | gcttcctttcgggc**ttaattaa**cagccggatctcagtgg | Quikchange oligos for altering the 3’ end of the M9 cassette in Pet29 to incorporate a PacI site. (Mod9-PacIend-R) |
| 131 | CAGACGATGAGCTG**TTAATTAA**TTCCCTAGCAAACATG | Mod 9-PacIF |
| 132 | CATGTTTGCTAGGGAA**TTAATTAA**CAGCTCATCGTCTG | Mod 9-PacIR |
| 209 | CGAGATCGATCCCGC**TTAATTAA**TACGACTCAC | QC1-M9-PacI-F |
| 210 | GTGAGTCGTA**TTAATTAA**GCGGGATCGATCTCG | QC1-M9-PacI-R |
| 211 | GGGGAATTGTGAGCGGA**TTAATTAA**TTCCCCTCTAGAA | QC2-M9-PacI-F |
| 212 | TTCTAGAGGGGAA**TTAATTAA**TCCGCTCACAATTCCCC | QC2-M9-PacI-R |
| 127 | CACCAGCACTACAGCCAACTGC | G06F |
| 128 | GACGTACGACCAGCTGCGATCACCACCGG | G06R |
| 129 | CACCGTACGGAGGAGCCGGTGGCGGTGGTG | F02F |
| 130 | GACGTACGTTAATTAATGGAGGTCTGTGATGACCTGT | F02R |
| 157 | CGCAGCTGGTCGTACGGAGGAGCCGGTGGCG | Quick-Change Mutagenesis (G06QC-F) |
| 158 | CGCCACCGGCTGGTCCGTACGACCAGCTGCG | Quick-Change Mutagenesis (G06QC-R) |
| 159 | ATACGACTCACTATAGGG | pcDNA2.1 T7 |
| 162 | GGTGGGTGTGGGGTGATCGAAGATGAGGGTGGGGGGCAGATCCAGGCCGGTGTT | M8-DEL-R |
| 163 | AACACCGGCCTGGATCTGCCCACCCTCATCTTCGATCACCCCACACCCACC | M8-DEL-F |
| 184 | gcgcatgccaagcgctgcgacagt | 703-F (MlsA2 / C1 sequencing) |
| 186 | gcgcatgcaatgttaaccggtggtgc | 705-F (MlsA2 / C1 sequencing) |
| 165 | GCGATGACGAGCAGGCGG | KSR1 (MlsA2 sequencing) |
| 167 | CTCGGTGAAAATCGCAGG | KSR2 (MlsA2 sequencing) |
| 164 | CTGCTGGAGGTGTGCTGG | KSF2 (MlsA2 sequencing) |
| 169 | GGCGTGGGTGATGGCTTG | KSR3 (MlsA2 sequencing) |
| 166 | AATCCTTACGCAACAACG | KSF3 (MlsA2 sequencing) |
| 168 | CCCTACACGCCACCTAC | KSF4 (MlsA2 sequencing) |
| 109 | CACCATGCGTCTGTACCAGCATCTCA | ATIF (MlsA2 sequencing) |
| 110 | ATAGTCAGGTGAGGCGAGTTG | ATIR (MlsA2 sequencing) |
| 18 | CTAGGCCACTCCATCGGAGAA | ACETATE_F (MlsA2 sequencing) |
| 32 | GGGAGGTGGTTGTGGTC | mlsBM7-14 (MlsA2 sequencing) |
| 31 | GGCCAGCTCAGGTGTAG | mlsBM7-13 (MlsA2 sequencing) |
| 30 | GTCGCTGTTGGTGTTGTC | mlsBM7-12 (MlsA2 sequencing) |
| 22 | CAGGACTGGCTCACTCAC | mlsBM7-04 (MlsA2 sequencing) |
| 29 | GCAGACAGGTTGTGTTCG | mlsBM7-11 (MlsA2 sequencing) |
| 28 | GCTGGGCTGATGCTTTC | mlsBM7-10 (MlsA2 sequencing) |
| 23 | TGCTCATGTCACCATCAC | mlsBM7-05 (MlsA2 sequencing) |
| 27 | GCCTGTGATGGCAGTTG | mlsBM7-09 (MlsA2 sequencing) |
| 24 | ACTGGCCCTGTTCGATG | mlsBM7-06 (MlsA2 sequencing) |
| 25 | TTTGAACCAAAGTTTCAGC | mlsBM7-07 (MlsA2 sequencing) |
| 26 | ACCGCCGAATACCAATAC | mlsBM7-08 (MlsA2 sequencing) |
| 77 | gggcaatcgtcctcactg | LM-F (Screen for 2-module MlsA1 PKS) |
| 78 | caagggcagtcttgattagg | LM-R (Screen for 2-module MlsA1 PKS) |
| 215 | CTCTGGACTTTTCCAACG | ER-F (screen for MlsA2 and sequencing of MlsA2) |
| 216 | CCTGGTCGAGTTGATCG | ER-R (screen for MlsA2 and sequencing of MlsA2) |
| 630 | GC**TCTAGA**ATGCTAGTCGCGGTTGATCGG | PermEF-XbaI (To PCR PermE-mup045-mup38 region from pCJW161 with XbaI ends) |
| 576 | GC**TCTAGA**CCTGACGCCCCAGTTCG | TEII-XbaI-R (To PCR PermE-mup045-mup38 region from pCJW161 with XbaI ends) |
| 81 | cagcaagtaacggtggaaca | 045-F (For PCR and RT-PCR screen) |
| 82 | acgtggcccatttgtcttag | 045-R (For PCR and RT-PCR screen) |
| 139 | ATTCAAACGGATGCGAACTG | 038-F (For PCR and RT-PCR screen) |
| 140 | ACATTGCTGGACAAACGACA | 038-R (For PCR and RT-PCR screen) |
| 175 | gttctcaccatgccagacct | crtI-F (For RT-PCR screen) |
| 176 | ggcgtagagggactggaag | crtI-R (For RT-PCR screen) |

^*^Introduced *SacII*, *EcoRV, XbaI, NdeI,* and *PacI* restriction enzyme sites are indicated in bold type.
